# High Definition Infrared Spectroscopic Imaging for Lymph Node Histopathology

**DOI:** 10.1371/journal.pone.0127238

**Published:** 2015-06-03

**Authors:** L. Suzanne Leslie, Tomasz P. Wrobel, David Mayerich, Snehal Bindra, Rajyasree Emmadi, Rohit Bhargava

**Affiliations:** 1 Beckman Institute for Advanced Science and Technology, University of Illinois at Urbana-Champaign, Urbana, Illinois, United States of America; 2 Department of Bioengineering, University of Illinois at Urbana-Champaign, Urbana, Illinois, United States of America; 3 Department of Electrical and Computer Engineering, University of Houston, Houston, Texas, United States America; 4 Department of Pathology, University of Illinois at Chicago, Chicago, Illinois, United States of America; 5 Department of Chemical and Biomolecular Engineering, University of Illinois at Urbana-Champaign, Illinois, United States of America; 6 Department of Electrical and Computer Engineering, University of Illinois at Urbana-Champaign, Illinois, United States of America; 7 Department of Mechanical Science and Engineering, University of Illinois at Urbana-Champaign, Illinois, United States of America; 8 Department of Chemistry, University of Illinois at Urbana-Champaign, Illinois, United States of America; Glasgow University, UNITED KINGDOM

## Abstract

Chemical imaging is a rapidly emerging field in which molecular information within samples can be used to predict biological function and recognize disease without the use of stains or manual identification. In Fourier transform infrared (FT-IR) spectroscopic imaging, molecular absorption contrast provides a large signal relative to noise. Due to the long mid-IR wavelengths and sub-optimal instrument design, however, pixel sizes have historically been much larger than cells. This limits both the accuracy of the technique in identifying small regions, as well as the ability to visualize single cells. Here we obtain data with micron-sized sampling using a tabletop FT-IR instrument, and demonstrate that the high-definition (HD) data lead to accurate identification of multiple cells in lymph nodes that was not previously possible. Highly accurate recognition of eight distinct classes - naïve and memory B cells, T cells, erythrocytes, connective tissue, fibrovascular network, smooth muscle, and light and dark zone activated B cells was achieved in healthy, reactive, and malignant lymph node biopsies using a random forest classifier. The results demonstrate that cells currently identifiable only through immunohistochemical stains and cumbersome manual recognition of optical microscopy images can now be distinguished to a similar level through a single IR spectroscopic image from a lymph node biopsy.

## Introduction

Fourier transform infrared (FT-IR) spectroscopic imaging uses molecular contrast measured by vibrational spectroscopy to create label-free images of biological samples [1–4]. Computer algorithms, further, process the acquired information into a visual format potentially usable by pathologists for clinical diagnoses, and research scientists for detailed molecular insight. Commercial FT-IR imaging instruments with 5.5–6.25 micron pixel sizes at the sample plane have provided data that can accurately reproduce some elements of tissue histopathology, for example for prostate [5–7], breast [8], and colon [9] biopsies through the use of machine-learning algorithms. These studies have been validated through comparison of the images to conventional clinical and research stains, clinical histologic diagnoses, disease stage, and patient recurrence [10–12]. The molecular information in the absorption spectrum acts as a unique vibrational spectroscopic “fingerprint” of the tissue at each pixel and is also intimately related to the sample morphology as well, while the spatial localization of the signal determines the level of detail accessible. Published reports have established the potential of IR imaging, but with pixel sizes significantly larger than those in optical microscopy, the morphologic visualization has not been comparable and blurring of different cell lines was likely. Recent progress in understanding image formation [13–15] and the development of high definition (HD) FT-IR spectroscopic imaging [13,16], provides a new opportunity to significantly increase the information content. However, no reports have yet examined tissue properties and the resulting images in detail. In particular, while HD imaging has promised significantly increased spatial detail and spectral localization, there has not yet been a report that quantitatively and objectively reproduces information provided by traditional histopathology techniques such as H&E and immunohistochemical (IHC) staining and flow cytometry.

Lymph node histology is especially challenging for traditional histopathology as well as for IR imaging. Lymph nodes are highly heterogeneous secondary lymphoid organs with a constant stream of lymphocytes, macrophages, and antigen presenting cells flowing through fibrovascular tissue enclosed by a fibrous capsule [17]. They are responsible for initiating immune responses, and as such are routinely used in diagnostic and prognostic evaluations for cancer and chronic inflammation or infection. IR spectroscopy has already proven capable of detecting subtleties between lymphoid tumors of different grades by quantifying changes in the absorbance ratio of nucleic acid peaks [18–22]. Early IR imaging work with cell lines, tonsil and spleen biopsies [2,23,24] showed that key lymphocytes such as T and naive B cells can be distinguished spectroscopically both in solution and by imaging biopsy sections. This is a remarkable result as these lymphocytes are morphologically identical in a visible H&E-stained image, and require IHC stains and/or flow cytometry to be distinguished by pathologists. Spectroscopic imaging then, with minimal sample preparation and without using labels or destroying the sample, is able to provide clinically useful information. This opens up the possibility of not only aiding pathologists in the clinic, for example through the distinction of B and T cell lymphomas, but also of providing a platform for fundamental research into the adaptive immune response in general. A full complement to clinical distinctions with cellular resolution, however, has been lacking, and identifying individual cells has not been possible.

More recently, subtypes of peripheral blood lymphocytes such as cytotoxic T cells, helper T cells, and naïve B cells were sorted using FT-IR imaging combined with Principal Component and Partial Least Square Discriminant Analyses (PCA and PLS-DA) [25]. The intricate and varied nature of lymphoid tissue and the large image pixel sizes of commercial FT-IR imaging instruments have restricted studies performed on lymph nodes themselves to descriptions of multi-cellular tissue morphology [26–29] and major changes in lymph nodes due to breast micro-metastases [30–33], typically through the use of Hierarchical Cluster Analysis (HCA) or Fuzzy C-means Clustering (FCM). For example, IR imaging data with 6.25 μm-sized pixels combined with HCA and PCA using the fingerprint region of the spectrum delineated the structure of the lymph node and various lymphocyte populations [34]. This pixel size, however, was on the order of the diameter of a typical lymphocyte, which left the resolution and classification of individual lymphocytes out of reach. While the larger image pixel size produced a high signal-to-noise ratio (SNR) and fast data acquisition times, it resulted in an averaging of spectra from different cells, limiting the distinction and classification of the heterogeneous lymphoid tissue. In this study, we sought to use HD IR imaging to resolve, distinguish, and classify naïve B cells, both dark zone and light zone germinal center B cells, T cells, red blood cells, connective tissue and the fibrovascular network, and smooth muscle in biopsies from normal, reactive, and malignant lymph nodes.

## Results and Discussion

There are three major components to the advances reported in this study. Instrumentation capable of providing rapid and high-quality HD data is discussed first. The suitability of the data quality for information extraction by algorithms is subsequently discussed and, finally, we seek to demonstrate whether the combination of data quality and specific analysis methods can provide visualizations of a sufficient quality.

**Fig 1:**
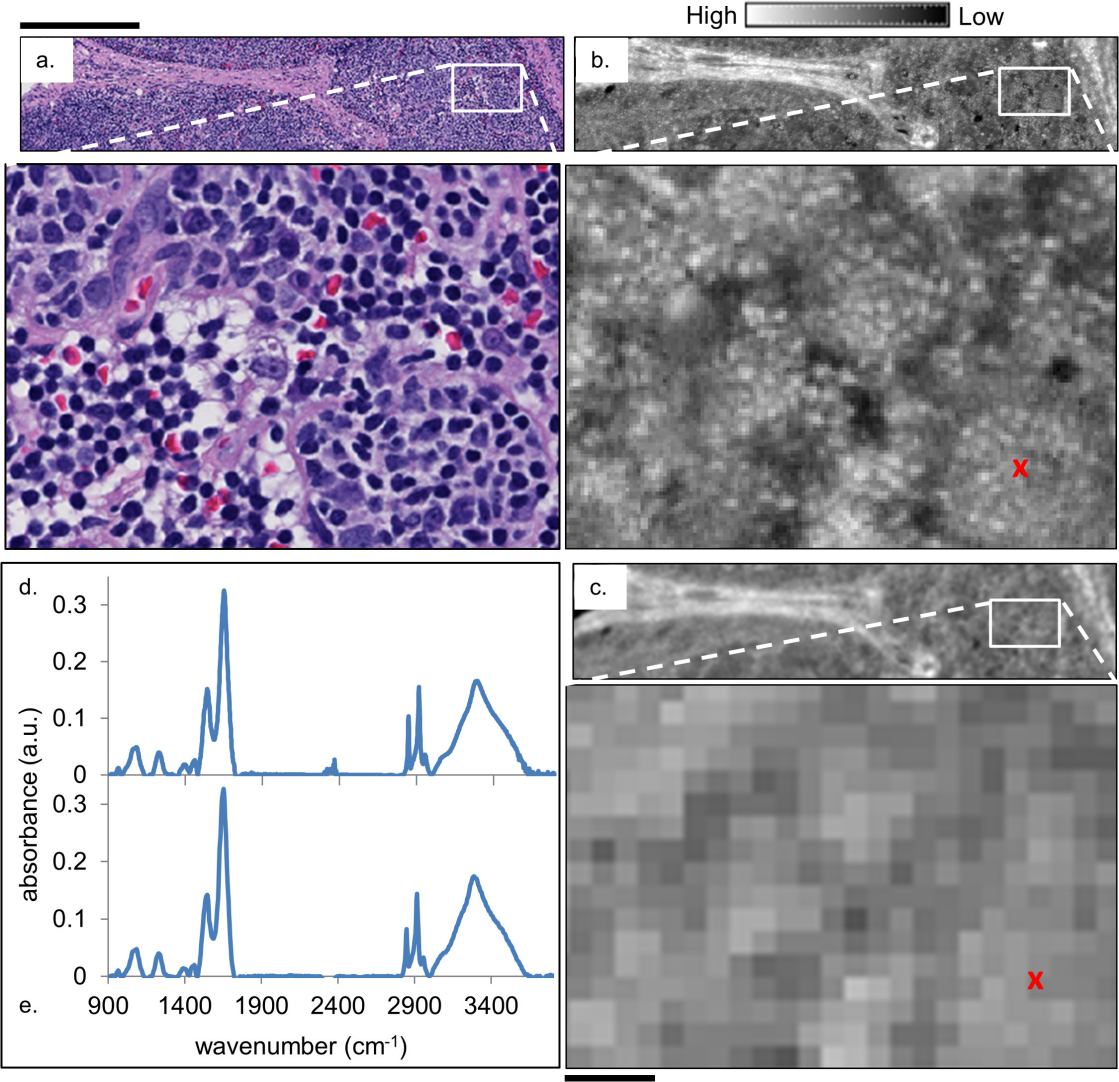
Comparison of H&E-stained optical microscopy and IR images of lymph node tissue. (a) H&E stained image from a healthy submandibular lymph node biopsy alongside (b) an HD IR image of a serial section of the lymphoid tissue and (c) the same region imaged with a lower resolution FT-IR instrument. The IR images show the absorbance at 3075 cm^-1^ after baseline correction. Sample spectra, (d) and (e), are plotted from the pixel marked with a red ‘x’ in (b) and (c), respectively. The black scale bar below the zoomed-in image in (c) corresponds to 50 μm, and above the H&E stained image to 300 μm.

We acquired data from samples and processed it as indicated in the methods section. In Fig 1, we compare the data quality obtained from conventional and HD instrumentation. An HD spectroscopic image (1.1 μm/pixel) from a healthy submandibular lymph node (Fig 1B) is presented along with the same region imaged using conventional IR imaging instrumentation (6.25 μm/pixel) and slightly lower numerical aperture (NA) objectives (Fig 1C). An H&E stained serial section of the biopsy is provided for reference in (a). While the high- and low-definition IR images both seem to be similar to the visible image, the benefits of HD imaging become apparent in comparing tissue regions in detail. Inspecting the magnified regions of the three images suggests that HD IR imaging may be able to distinguish the individual lymphocytes that are clearly apparent in the H&E images and that appear only as unrecognizable pixels in the conventional low-definition IR image that is similar to previous reports. Sample spectra from approximately the same location in one of the lymphocyte clusters (indicated by a red ‘x’) in the HD and low resolution images are presented in Fig 1D and 1E, respectively. It can be clearly seen that the increased contrast and image quality from HD imaging and its associated processing does not result in a compromise in spectral quality. It is notable that such high performance was achieved with a desktop system, since this has only previously been reported for microscopy with high intensity sources such as a synchrotron [35] or quantum cascade laser [36,37]. It is this increase in spatial and spectral specificity that has the potential to make individual lymphocyte classification attainable. To achieve recognition of cells, numerical algorithms must be employed.

The technological advance in finer pixel size demonstrated in Fig 1 brings with it new challenges. The first challenge is one of data quality. Since in HD imaging the same signal is distributed across more pixels in the detector, a reduction in SNR is observed. Assuming that the intensity of the source and the integration time of the detector have already been optimized, the SNR can be improved in two other ways: by increasing the number of co-additions for a given image, which increases the data acquisition time, and/or by performing noise reduction techniques such as Minimum Noise Fraction (MNF)-based noise rejection after collecting the data [38–41]. Both techniques were employed for the HD IR data presented in Fig 1 and throughout this paper.

The second potential challenge arises in the classification of the HD images since the IR absorption spectrum from a single pixel can now correspond to subcellular features instead of a whole cell or an average of several adjacent cells. Prior IR spectroscopic imaging of lymphoid tissue has been performed using large image pixels with subsequent pixel binning before attempting chemometric evaluation [30–32]. Fig. A in S1 File compares a secondary follicle from a healthy lymph node imaged in both HD and low resolution and classified using Hierarchical Cluster Analysis (HCA), a popular approach for initial data analysis frequently used to identify structures in lymphoid tissues [23,30,31]. In general, applying HCA to HD images (Fig. Ac in S1 File) increases the image detail and the total number of image pixels, but the presence of subcellular spectral contributions complicates the analysis. While HCA may be successful for large pixel sizes due to an entire cell occupying each pixel, it seems that naïve methods such as clustering may not work well when pixel contributions can arise from subcellular domains that have distinctly different spectral signatures than the average across the whole cell. As a general discovery tool, it appears that conventional unsupervised methods may not be compatible with subcellular pixel sizes. The most fundamental limitation of clustering techniques in HD FT-IR is the factor that is normally their strength – the dataset division due to variance sources. In the finest detail images (Figs Ac, Bf, Ca, and Cc in S1 File), clusters that do not correspond to a particular cell type but rather to subcellular regions that are similar across classes are seen. In HD FT-IR there are clearly new subcellular spectral contributions that can confound clustering results and may limit its applicability to distinguishing lymphocyte populations and recognizing tissue morphology.

To overcome this hurdle, we developed a supervised classifier using the Random Forest algorithm [42] to take full advantage of both the high spatial and spectral quality of the HD spectroscopic images. To maintain full spatial detail, no pixel averaging is performed, and to maximize accuracy, information across the full mid-IR spectrum of the tissue is included. This ensures that the information captured by the subcellular image sampling is preserved, and that all biologically-relevant spectral features that may aid in classification are utilized. Furthermore, while HCA yields an image-specific result which cannot be easily transferred to other images, classification schemes work on every pixel independently and are therefore free of this limitation. Once trained, a random forest classifier can be applied to the classification of any subsequent image.

**Fig 2:**
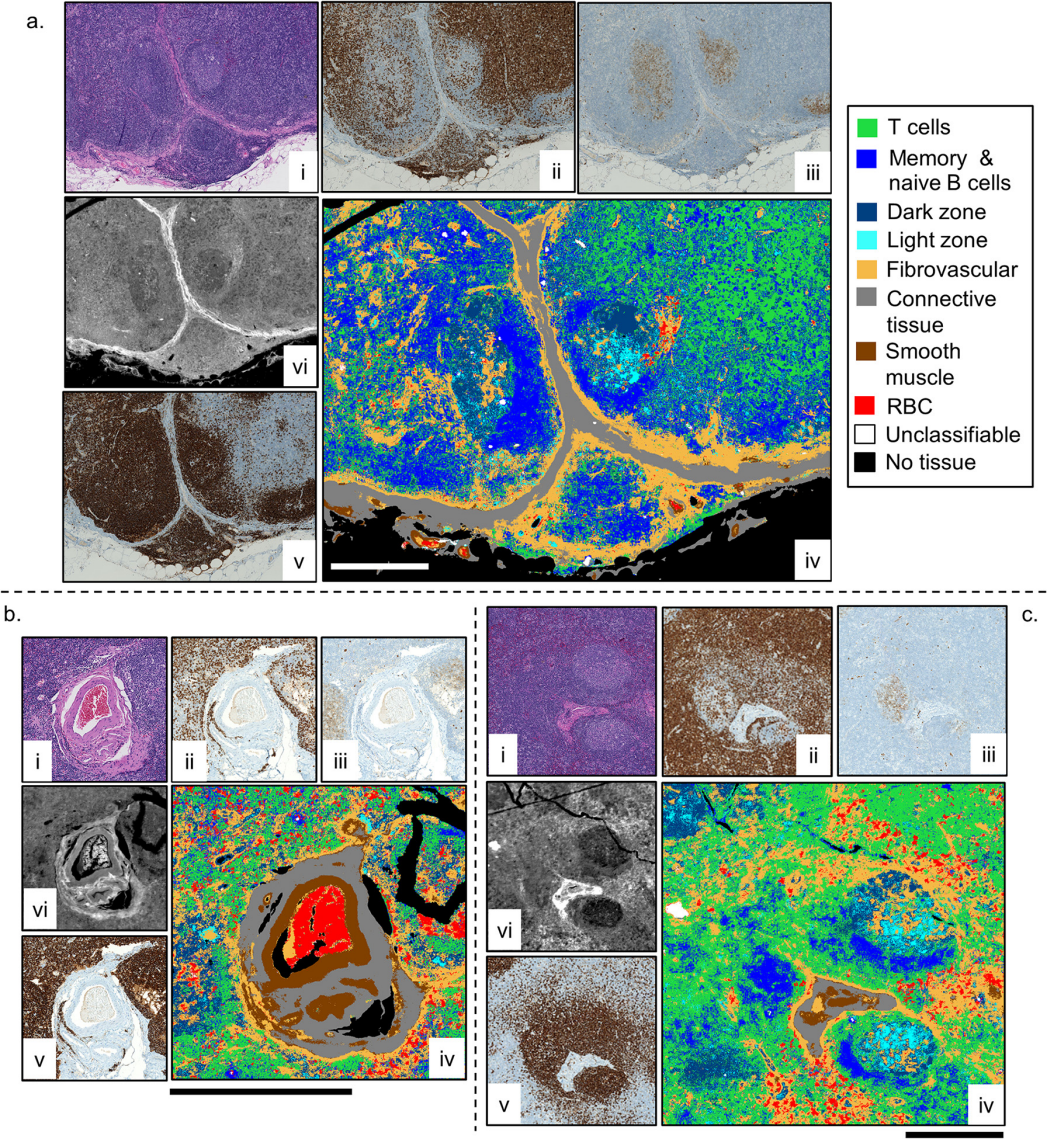
HD training and classification using three demonstrative examples. For each sample (a-c), H&E (i) and IHC stained serial sections CD3+ (ii), CD10+ (iii), and CD20+ (v) were used to draw ROIs on the HD IR images, (vi), to train the classifier. The resulting classified images, (iv), are shown. The absorbance for the IR images is at 3300 cm^-1^, and the solid bar in each classified image is 500 μm.

In order to be able to fully represent the structure of the lymph node as well as its cellular populations as determined by a board-certified pathologist, nine classes were chosen: naïve and memory B cell, T cell, activated B cell—dark zone, activated B cell—light zone, red blood cells, connective tissue, fibrovascular network, smooth muscle, and “other” or unclassifiable. The unclassifiable class was used to represent regions in the tissue corresponding to debris or other confounding components not related to the expected cell types in a lymph node. Three example images from a healthy submandibular lymph node that clearly exhibited these classes in the H&E and IHC stained images were selected to train the classifier, and are shown in Fig 2. In each of these examples, the contrast in the IR images is comparable to that displayed in the accompanying molecular staining images. Different types of lymphocytes cannot be distinguished easily in the H&E images, (i), due to the limitation of morphology in performing phenotype separation. The IHC images, (ii,iii,v), highlight the locations of lymphocytes with CD3+, CD10+, and CD20+ cellular receptors, respectively, but require multiple sections and time-intensive manual investigation by optical microscopy. Furthermore, concerns about staining variability and the non-quantitative nature of IHC often limits the visualization. With IR chemical imaging, individual cells can be observed and different cell types can be discerned by displaying different bands in the mid-IR absorption spectrum of the tissue. The accuracy limit of IR images depends on the SNR of the data as well as the spectral purity at each pixel. With the high SNR and high spatial localization in HD imaging, the capability of IR imaging is approaching that of IHC staining and optical microscopy.

The H&E and three IHC (CD3+, CD10+, CD20+) stained serial sections were used under pathologist supervision to draw regions of interest (ROIs) corresponding to the nine classes onto the three training images (see S1 Fig). A tissue mask was applied to each image that limited the classification to those pixels with an absorbance value greater than 0.2 at the Amide I band (1654 cm^-1^). Those pixels beneath this absorbance threshold appear “black” in the classified images. Following the established methods of selecting metrics from the spectrum [6], the training and subsequent classification of each HD data set was performed in minutes, scaling with the total number of pixels and trees. The resulting classification of the three training images is shown in Fig 2 (iv), and demonstrates a close resemblance to the H&E (i) and IHC images (ii, iii, v) in each case. Note that Fig 2C is the same HD IR image that was analyzed by HCA with different pixel averaging in Fig. B in S1 File. Comparison of the supervised and unsupervised algorithms shows that the supervised results better resemble images used in the clinic.

**Fig 3:**
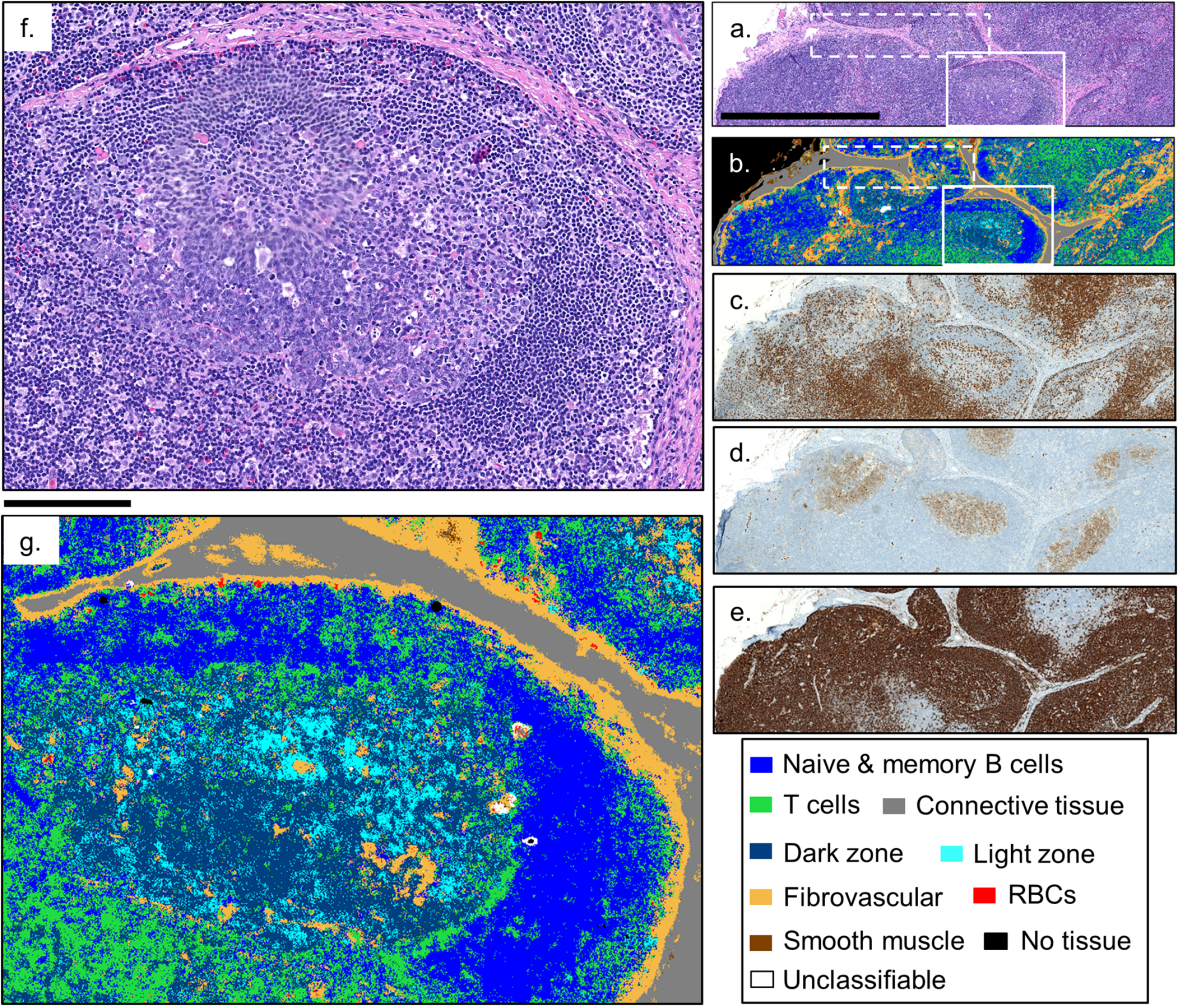
Classification of an HD IR image of a normal submandibular lymph node. A qualitative comparison of the classified HD IR image, (b), can be made with the stained serial sections (a) H&E, (c) CD3+, (d) CD10+, and (e) CD20+, showing excellent agreement. Zooming in on the secondary follicle in the HD classified image, (g), shows that the classifier is capturing the naïve B cell mantle (blue), T cells (green), and the light and dark zone regions of activated B cells (cyan and dark blue, respectively) of the germinal center. The dashed boxes correspond to the images shown in Fig 1. The solid bars in (a) and (f) are 1 mm and 175 μm, respectively.

While previous results have demonstrated the capability to recognize specific cell populations, the accuracy and speed for complex image segmentation in intact lymph node sections was not probed. A healthy lymph node consists of three zones: the cortex, the paracortex, and the medulla. Each of these three regions has its own structure and respective lymphocyte populations that change when an immune response is initiated. As Fig 3 shows, our random forest classifier can, in minutes, classify a 1 mm x 3 mm HD IR image, and not only correctly render the large scale structures such as the connective tissue (grey) of the trabeculae and capsule, and fibrovascular framework (orange) of the cortex and paracortex, but zooming in on the boxed region (Fig 3G), shows that the classifier accurately captures the finer features such as the light and dark zones (cyan and dark blue, respectively) of a germinal center as well [43]. The dashed boxes in Fig 3A and 3B indicate the regions shown in Fig 1A and 1B).

T cells serve to activate naïve B cells in the presence of specific antigens, and are predominantly found in the cortex just deep to the capsule and in the paracortex that surrounds primary and secondary follicles [17]. A CD3+ IHC stain, shown in Fig 3C, of a serial section of the lymph node biopsy can be used to confirm the appropriate distribution of T cells (green) in the classified image, and shows excellent qualitative agreement. Naïve B cells are typically localized in primary and the mantle zones of secondary follicles. A CD20+ stained serial section, shown in Fig 3E, helps distinguish naïve and memory B cells (which stain brown) from the morphologically identical T cells. Comparing Fig 3B, 3C and 3E confirms that the HD lymphoid classifier successfully represents and distinguishes between these important lymphocyte classes throughout the cortex and paracortex. Zooming in on the secondary follicle boxed in Fig 3B shows that, even with subcellular sampling, the HD classifier is able to distinguish and classify the lymphocyte populations. Fig 3G shows an appropriate naïve B cell population (blue) comprising the mantle zone, and a typical distribution of T cells (green) in the germinal center as is expected from their role in B cell activation and is confirmed in the CD3+ stain in (c). The ability to classify T (green) and naïve B (blue) cells by their IR absorption spectrum, as demonstrated previously [23,25], has particular clinical relevance due to the inability to distinguish these lymphocytes by morphology in the H&E stained visible images in Fig 3A and 3F). The CD10+ stained serial section, shown in Fig 3D, indicates the locations of the activated B cells that populate the germinal centers and also natural killer cells [43], and shows good correspondence with the light and dark zone classes in Fig 3B and 3G).

The spectroscopic distinction between the activated B cells of the light (cyan) and dark (dark blue) zones of the germinal center is a novel result that, to our knowledge, has not been realized before with IR spectroscopy, suggesting the potential for HD spectroscopic imaging and classification to be applied to ongoing research into the role of germinal center and activated B cells in B-cell lymphomas [43–45]. These HD classification results represent the first time that individual cells in tissue as heterogeneous as lymph nodes have been classified by either supervised or unsupervised means. Additional classified HD IR images of the healthy lymph node can be found in the supplementary material (S2–S4 Figs) along with the spectral metrics for classification obtained in our approach (S1 Table).

**Fig 4:**
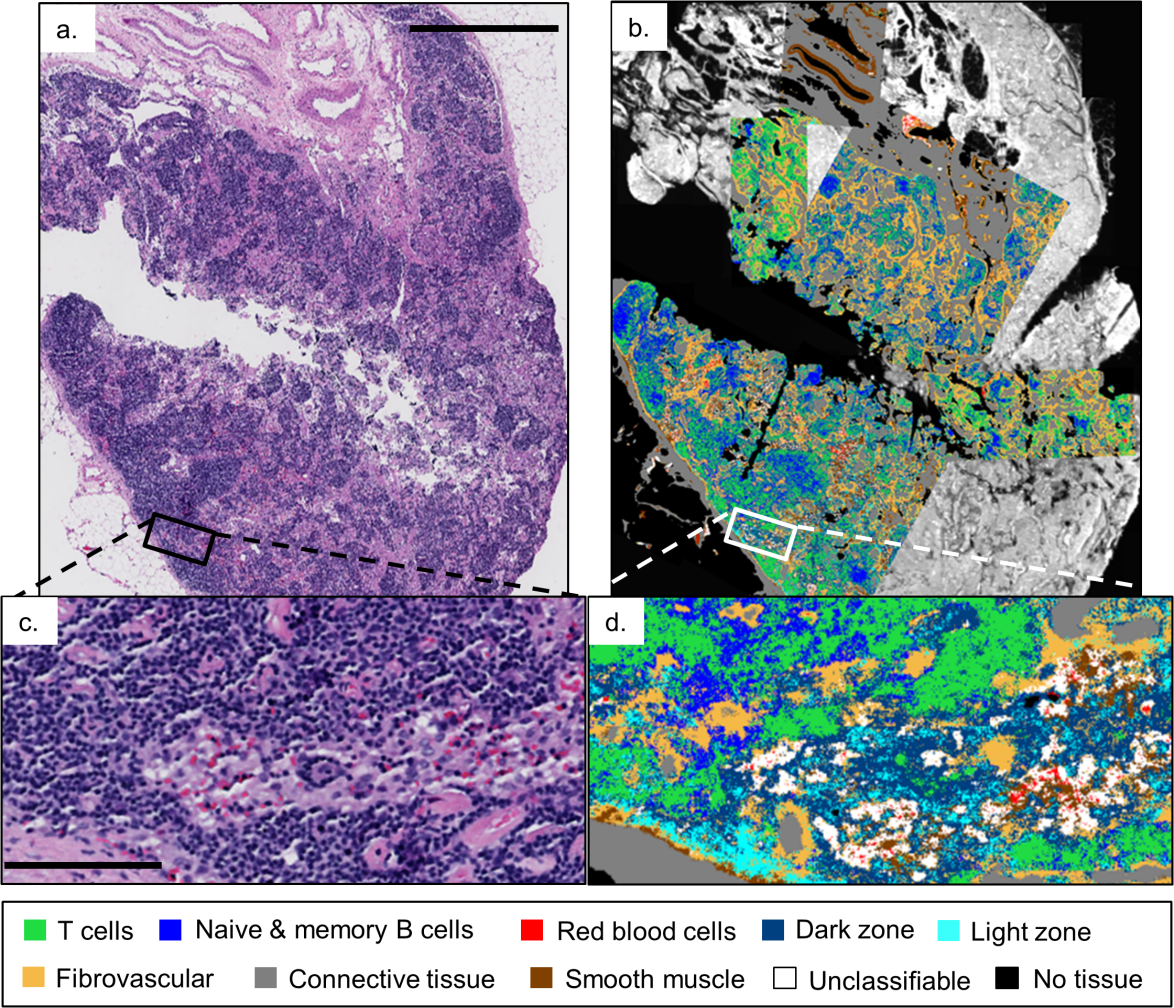
Classification of HD IR images from a reactive iliac lymph node. (a) H&E stained serial section presented for comparison to HD classification images overlaid on a low-resolution 4cm x 2cm IR image displaying the intensity of the 1654 cm^-1^ band, (b). The zoom-in, (d), of the white box in (b) shows the classification of RBCs, T cells, naïve and memory B cells, along with the light and dark zone activated B cells in the sinusoid. The black bars in (a) and (c) are 1.5 mm and 125 μm, respectively.

While Fig 3 demonstrates the potential of HD IR imaging for studying normal lymph node structure and lymphocyte population distributions, we further examined whether novel information can be obtained from a reactive lymph node. When an immune response is initiated, the lymph node reacts by forming secondary follicles that expand to fill the cortex, and medullary sinuses that pass T cells, activated B cells, and macrophages to the medulla of the lymph node [17]. We first sought to examine whether our random forest classifier that was trained on a healthy submandibular lymph node could be applied to a reactive iliac lymph node. Several regions of a serial section of the lymph node shown in Fig 4A were imaged in the IR. Fig 4B shows an overlay of six individually classified HD IR images on a low-resolution IR image of the biopsy section with the intensity at the 1654 cm^-1^ band.

Zooming in on one of the classified images in Fig 4B, shows the characterization of a medullary sinus filled with RBCs (red), T cells (green), and light and dark zone activated B cells (cyan and dark blue, respectively) moving in and out of the fibrovascular network (orange) and through the connective tissue (grey) that provides the underlying structure of the lymph node [17]. The blue class, while perhaps seemingly over-represented in a reactive lymph node, captures both naïve and memory B cells, highlighting the multi-faceted role of B cells throughout an immune response.

**Fig 5:**
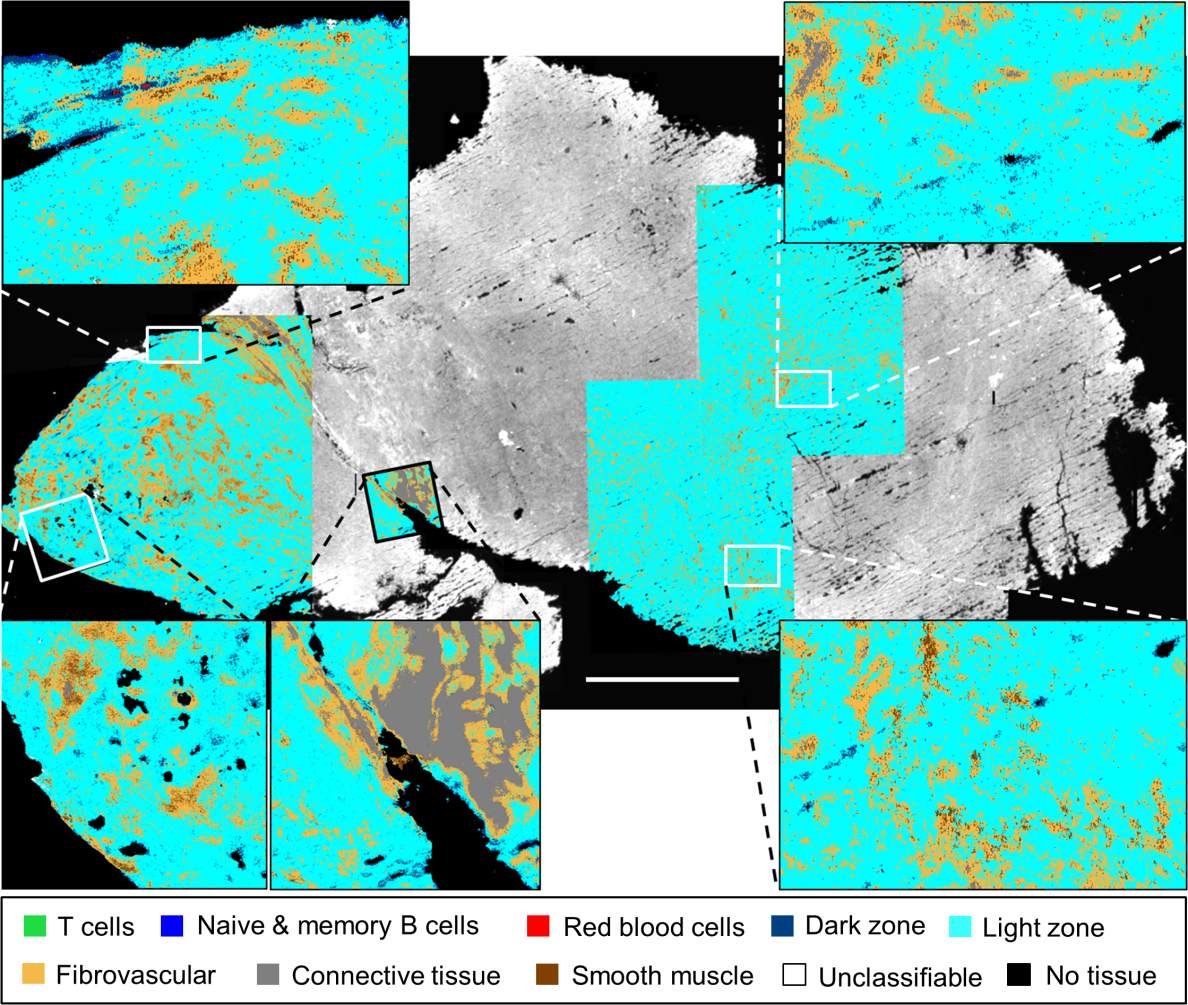
Classification of HD IR images from a malignant mesenteric lymph node. Five individually classified HD IR images overlaid on a low-resolution IR image of an abnormal lymph node biopsy with the band intensity at 1654 cm^-1^. The zoomed-in regions clearly show consistent activated B cell (more specifically, light zone) classification, in agreement with the diagnosis of diffuse, large B-cell lymphoma made by flow cytometry. The white bar is 2 mm.

Due to the difficulty in distinguishing B and T cells, the diagnosis and grading of many types of lymphoma are typically determined via flow cytometry or a complicated and time consuming series of IHC stains performed on serial sections of the biopsy [46]. Flow cytometry from a serial section of the abnormal mesenteric lymph node biopsy in Fig 5 was used to detect the presence of a clonal B-cell population expressing CD45 (leukocyte common antigen), CD19 (a B cell lineage marker), and CD22 (a B-cell specific antigen) leading to the diagnosis of diffuse, large B-cell lymphoma (DLBCL) by the surgical pathologist [45,47,48].

Remarkably, the classified HD images shown in Fig 5 clearly agree with this diagnosis even though the classifier was trained on healthy lymphoid tissue. The five independently classified HD IR images show consistent mature B cell classification across the 1.5 cm x 1 cm biopsy. Inspecting the zoomed-in boxes confirms that even though the sheets of cancer cells have effaced the lymph node, the HD classifier recognizes the individual lymphocytes as being of B cell origin, and more specifically as belonging to the light zone (cyan) class of activated B cells [43,44]. Interestingly, the dark zone class (dark blue), like the light zone class, is also comprised of activated B cells found in the germinal center of healthy lymph nodes, but it is only the fourth most populous class represented in the images after light zone, fibrovascular network (orange), and connective tissue (grey). This level of B cell classification specificity by the random forest classifier was surprising and suggests future studies on the potential of HD IR classification to detect different molecular subtypes of B cell lymphoma [44,45].

**Fig 6:**
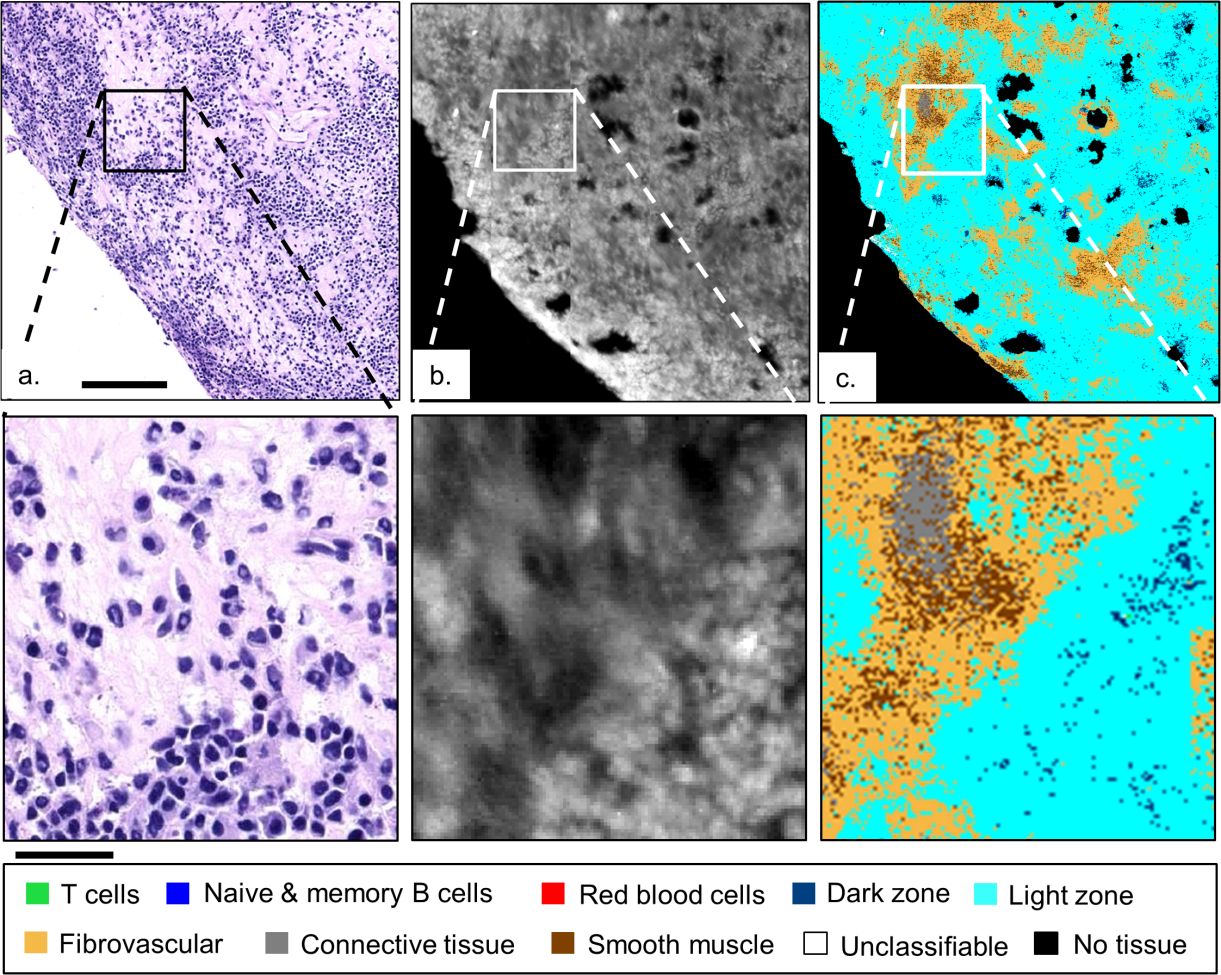
Comparison of malignant lymphoid tissue. H&E stained serial section (a), HD IR image at 3300 cm^-1^ intensity (b), and classified image, (c), from Fig 5 (bottom left) confirm the resolving power of HD spectroscopic imaging and the consistent light zone B-cell classification of the abnormal lymph node. The solid bar in (a) and below the zoom are 140 and 35 μm, respectively.

Fig 6 focuses on the bottom left HD classified image in Fig 5, comparing it to an H&E stained serial section and to the HD IR image with the intensity at the 3300 cm^-1^ band. Fig 6B and 6C) demonstrate both the ability of HD spectroscopic imaging to resolve individual malignant B cells, and that the random forest classifier can consistently classify the abnormal lymphocytes in spite of subcellular pixel sampling and having been trained on healthy lymphoid tissue. Further, the light zone (cyan) and fibrovascular network (orange) classes are dominant and consistent with the diagnosis of diffuse, large B-cell lymphoma. The presence of the smooth muscle class (brown) in Fig 6C, suggests a possible confusion of the classifier between the spectral contributions of smooth muscle from the regions of healthy lymph node tissue that were used to train the classifier (see S1 Fig), and the connective tissue of the malignant lymph node possibly due to hyalinization [48].

We want to emphasize that a comprehensive histopathologic enumeration was not the focus of this paper. Here we only seek to show the improvement in image quality leading to previously impossible cellular recognition. A more detailed study is needed for comprehensive histologic classification that may include cells such as macrophages, plasma cells, and dendritic cells, as well as a detailed analysis of classifier performance for cells in normal and diseased states. While we have divided the tissue cells into sub-classes of connective tissue, as an example, the histologic distribution of these samples is more complicated. The smooth muscle class seems more prominent that might be expected in the reactive and malignant classified images. The smooth muscle class was created from regions of interest (ROIs) immediately surrounding the blood vessel in Fig 2B (ROIs shown in S1B Fig) and is a very robust class for the healthy tissue. While "fibrovascular," "connective tissue," and "smooth muscle" are distinct and robust classes in the healthy lymph node images, it is not surprising that there might be classifier confusion in the reactive and especially the malignant lymph node images where the type and chemical composition may differ significantly from the healthy tissue. Thus, a comprehensive histologic analysis of lymph nodes will have to consider both cellular variances and disease relationships.

Our application of HD spectroscopic imaging to the classification of lymphocytes and tissue in lymph nodes confirms the exciting potential of this powerful new technology to provide complementary information to pathologists, and also highlights the possibility of applying these techniques to other key cells in lymph nodes such as plasma cells, dendritic cells, and macrophages, along with studies of tumor-infiltrating lymphocytes (TIL) in other tissues [25,49]. As a key measure of immune response, TIL have recently been shown to be a highly relevant prognostic marker in breast cancer [49–51], and are a promising area for future HD spectroscopic lymphocyte work.

## Conclusions

HD IR imaging of lymphoid tissue with unprecedented spatial and spectral quality is reported in this manuscript. While previous reports [52] on HD IR imaging have focused on instrumentation and theory with example images from tissue, to our knowledge, this is the first report of HD imaging and comprehensive classification for any tissue. The micron-sized pixels allow for subcellular sampling and the resolution of individual lymphocytes, and have caused us to re-examine conventional tissue classification techniques. Data from a healthy lymph node was used to construct a nine class, HD random forest lymphoid tissue classifier that provides the spatial and tissue detail that is needed for lymph node analyses. Various histologic structures as well as individual lymphocytes can be quickly and accurately classified using information distributed across the entire mid-infrared absorption spectrum. Salient features of both reactive and abnormal lymph nodes could be easily discerned, reproducing the key lymphocyte classes and even agreeing with the diagnosis of a diffuse, large B-cell lymphoma. The ability to resolve, distinguish, and classify individual lymphocytes in tissue opens many exciting avenues of future histopathological lymphocyte work including extension to the classification of plasma cells, macrophages, and antigen presenting cells, investigating and characterizing the immune response via tumor-infiltrating lymphocytes in breast tissue [50], and the role of activated and germinal center B cells in the development and molecular sub-typing of B cell lymphomas [44].

## Methods

### Ethics

The work presented here was performed on diagnostic specimens with information that neither identified the subjects directly nor indirectly through identifiers linked to the subjects. It was approved by and performed in accordance with the University of Illinois at Urbana-Champaign Institutional Review Board. The approved project is entitled “Optical spectroscopy and imaging of archival fixed tissue,” case number 06684, and consisted only of secondary analysis performed on anonymized archival tissue and, as such, according to the University of Illinois at Urbana-Champaign IRB policy, is exempt from written, informed consent.

### Samples and preparation

All tissues were obtained from the archives of the department of Pathology, University of Illinois Hospital & Health Sciences System. These tissues were selected, anonymized, and tissue sections supplied with limited clinical information by our pathologist Dr. Emmadi. The information given for the 3 cases are as follows:

Case 1: Normal submandibular lymph node incidentally removed from a 58 year old female with benign salivary gland issues, otherwise healthy.

Case 2: Benign, reactive iliac lymph node (sinus histiocytosis) taken from a 22 year old female who had undergone kidney transplant for non-tumor kidney disease.

Case 3: Malignant mesenteric lymph node, diagnosed as diffuse large B-cell lymphoma by flow cytometry, taken from a 72 year old female with a previously diagnosed diffuse large B-cell lymphoma.

Six micron-thick biopsy sections from the above three cases were placed on separate BaF_2_ slides and deparaffinized for 48 hours in a hexane bath. Serial sections were placed on glass slides and stained with Hematoxylin and Eosin (H&E). For the healthy lymph node, the following additional immunohistochemical (IHC) stains were performed on serial sections: CD3+, CD10+, and CD20+ [47,48]. All glass slides were imaged at 40x magnification with a NanoZoomer Digital Pathology System.

### Data acquisition

The HD FT-IR data were collected in transmission mode with a spectral resolution of 4 cm^-1^, an undersampling rate (UDR) of 4, and 90 μs integration time using an Agilent Stingray imaging system in “high-magnification” mode with a 680-IR spectrometer coupled to a 620-IR imaging microscope with a liquid nitrogen cooled mercury cadmium telluride (MCT) 128 × 128 focal plane array (FPA). All data collection and initial processing were performed using the Resolutions Pro software. For each 128 x 128 pixel image tile, 32 interferograms were averaged, ratioed against a 120 co-addition background transmission image, and processed with a triangular apodization function. The resulting interferogram was Fourier-transformed and the spectrum was truncated to the 900 – 3800 cm^-1^ spectral range. To produce an individual 128 x 128 pixel absorbance image tile described above, corresponding to 140 microns x 140 microns on the sample, required approximately 2 minutes of scan time and a subsequent 30 seconds of processing. The “high magnification” mode uses matched NA = 0.62 (numerical aperture) objectives and a secondary 5x magnification stage before the detector, resulting in an image pixel size of 1.1 microns.

Each HD spectroscopic image tile was then imported into ENVI 4.8, mosaicked using in-house software, and noise-reduced using the ENVI Minimum Noise Fraction (MNF) protocol [38,40,41] before being converted to metrics files using 11 metric definitions spanning the 980 – 3000 cm^-1^ spectral range (see S1 Table). The metrics-reduced images were then imported into Matlab (R2011b) and classified using a random forest algorithm with nine classes and 40 trees.

The low-resolution FT-IR data were collected in transmission mode on a Perkin-Elmer Spotlight 400 FT-IR imaging system with a Spectrum One spectrometer and a 16 element liquid nitrogen cooled mercury cadmium telluride (MCT) Linear Array (LA) detector equipped with an external dewar. The interferograms were the average of 16 scans per pixel, taken at a spectral resolution of 8 cm^-1^, and ratioed against a 120 scans per pixel transmission background spectrum. The spectra were truncated to the 750 – 4000 cm^-1^ range and atmospheric correction was performed along with other proprietary processing in the Spectrum Image software. The Spotlight 400 FT-IR imaging system uses matched NA = 0.6 objectives and has a minimum image pixel size of 6.25 microns. Individual regions of interest spanning the lymph node biopsies were imported into ENVI 4.8 and mosaicked.

### Algorithms and Processing

Hierarchical Cluster Analysis (HCA) was performed using Cytospec 2.0 64-bit software. Spectra were previously noise-reduced using the Minimum Noise Fraction (MNF) method in ENVI 4.8, then second derivatives were calculated using the Savitzky-Golay algorithm with 13 smoothing points. Finally, before performing HCA over a given spectral range, the data were vector normalized in that range. HCA was performed in 940–1265 cm^-1^ range with either Euclidean or D-Values (based on Pearson’s correlation coefficient) distances.

## Supporting Information

S1 Fig Regions of Interest (ROIs) for classifier training. ROIs for each of the nine classes were drawn onto each of the three HD IR training images (a-c), shown here with absorbance at 3300 cm^-1^. The solid white bars are 500 microns in each HD IR image.
(TIF)

S2 Fig Classification of HD FT-IR spectroscopic image of a 1 mm x 0.5 mm section of normal lymph node. A qualitative comparison of the classified HD IR image, (b), can be made with the stained serial sections (a) H&E, (c) CD3+, (d) CD10+, and (e) CD20+, and shows excellent agreement. The solid bar in (a) is 200 microns.
(TIF)

S3 Fig Healthy lymph node classification. Eight classified HD FT-IR images of a healthy submandibular lymph node overlaid on a low-resolution FT-IR image at 1654 cm^-1^ band intensity, (a), and compared to serial sections of H&E, (b), CD3+, (c), CD10+, (d), and CD20+, (e), stains show that the classifier captures the global structure of the lymph node. The classified images with black outlines in (a) contain ROIs that were used to train the classifier (see Fig 2 and S1 Fig). The solid bar is 1 mm.
(TIF)

S4 Fig Healthy lymph node classification. Seven classified HD FT-IR images of a healthy submandibular lymph node overlaid on a low-resolution FT-IR image at 1654 cm^-1^ band intensity, (a), and compared to serial sections of H&E, (b), CD3+, (c), CD10+, (d), and CD20+, (e), stains show that the classifier captures the global structure of the lymph node. The boxed classified image in (a) contains ROIs that were used to train the classifier (see Fig 2 and S1 Fig). The bar in (b) is 2 mm.
(TIF)

S1 File Fig. A. Comparison of HCA performed on HD and low-res IR images. A secondary follicle in a healthy lymph node was imaged with an HD FT-IR instrument, (a), and a conventional system, (b). Corresponding HCA results with 10 classes are shown in (c) and (d), respectively. IR images are of 2900 cm^-1^ band intensity as indicated by the bar, and the solid bar is 200 microns. **Fig. B. Effect of pixel binning on HCA.** HCA results with 10 classes performed on the same HD IR image from a healthy submandibular lymph node at different levels of pixel averaging: (a) 20x, (b) 10x, (c) 5x, (d) 3x, (e) 2x and (f) 1x (original HD image). The solid bar is 400 microns. **Fig. C. Binning effects on HCA.** HCA results with 10 classes and 2x binning, performed on the image shown in Fig. A in S1 File with two different distance measures: D-Values and Euclidean located on the top and bottom rows, respectively. The left column is the original HD data, while the right column presents results from 2x-binned HD data. The solid bar is 200 microns.
(DOCX)

S1 Table Eleven metric definitions used in the random forest classifier. (TIF)
