# Supplementary figures and images for: High Definition Infrared Spectroscopic Imaging for Lymph Node Histopathology
Source: PLoS One. 2015 Jun 3;10(6):e0127238. doi: 10.1371/journal.pone.0127238 (PMC4454651; doi:10.1371/journal.pone.0127238)

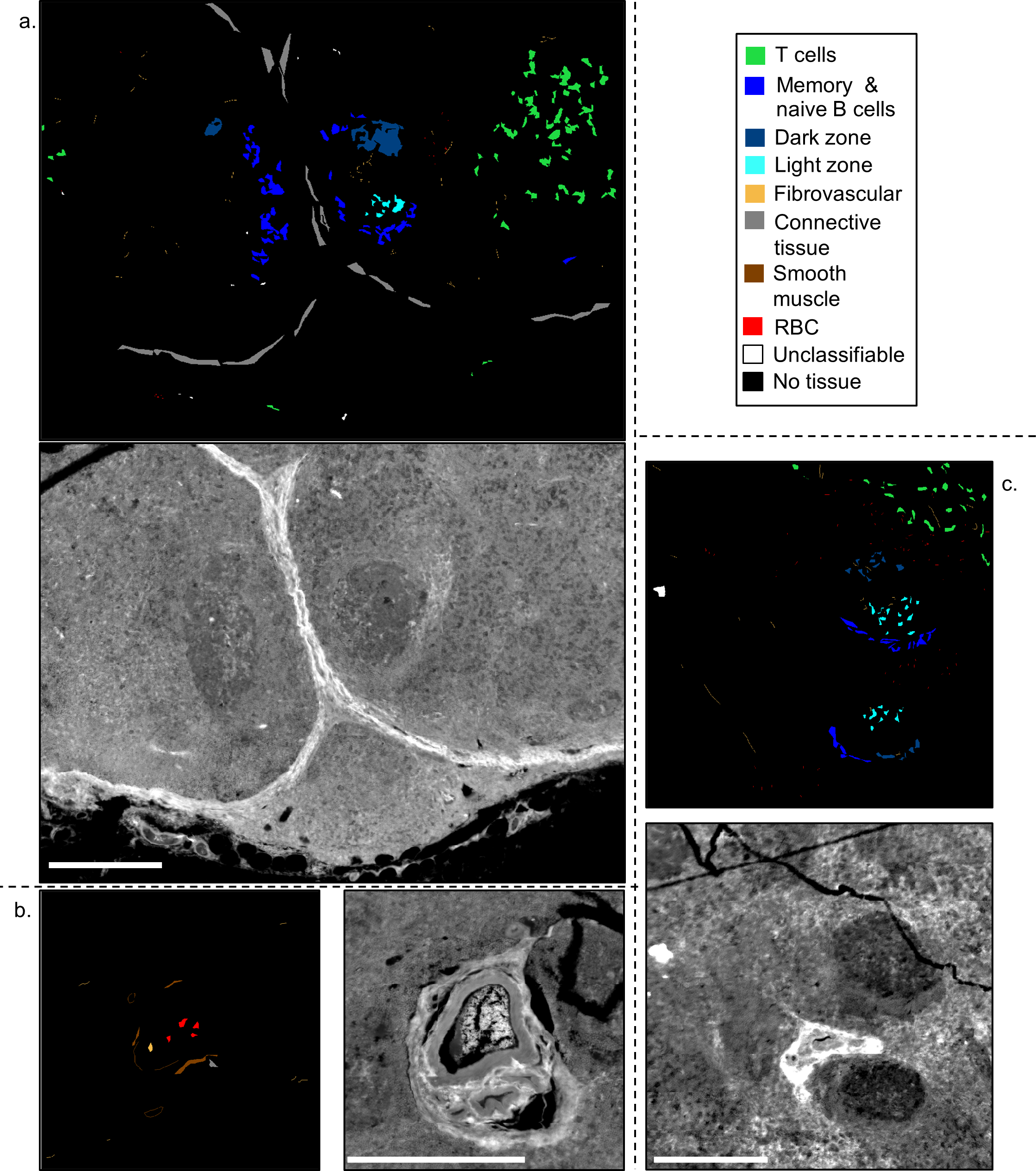

Supplement: S1 Fig — ROIs for each of the nine classes were drawn onto each of the three HD IR training images (a-c), shown here with absorbance at 3300 cm-1. The solid white bars are 500 microns in each HD IR image. (TIF) [file pone.0127238.s001.tif]

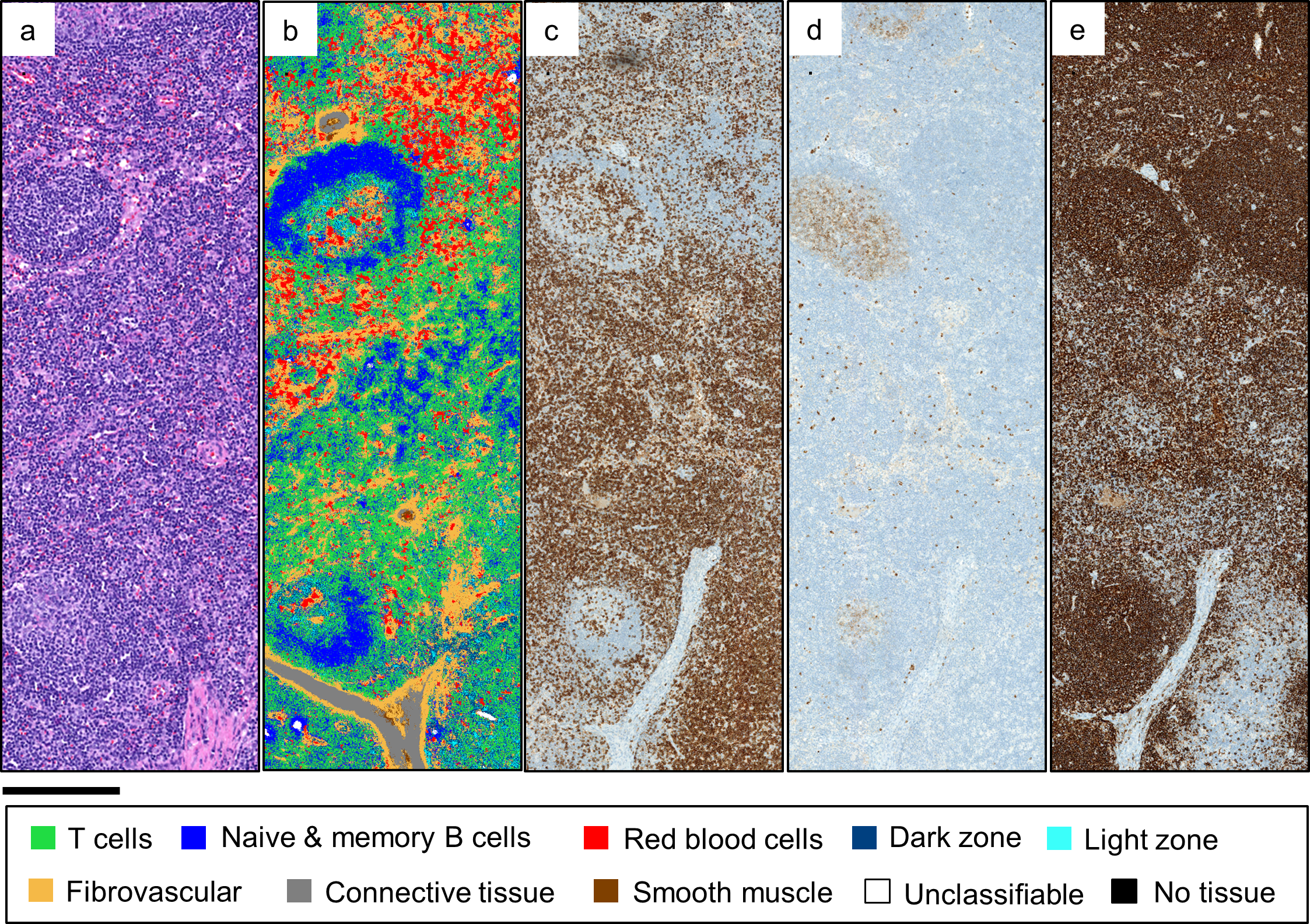

Supplement: S2 Fig — A qualitative comparison of the classified HD IR image, (b), can be made with the stained serial sections (a) H&E, (c) CD3+, (d) CD10+, and (e) CD20+, and shows excellent agreement. The solid bar in (a) is 200 microns. (TIF) [file pone.0127238.s002.tif]

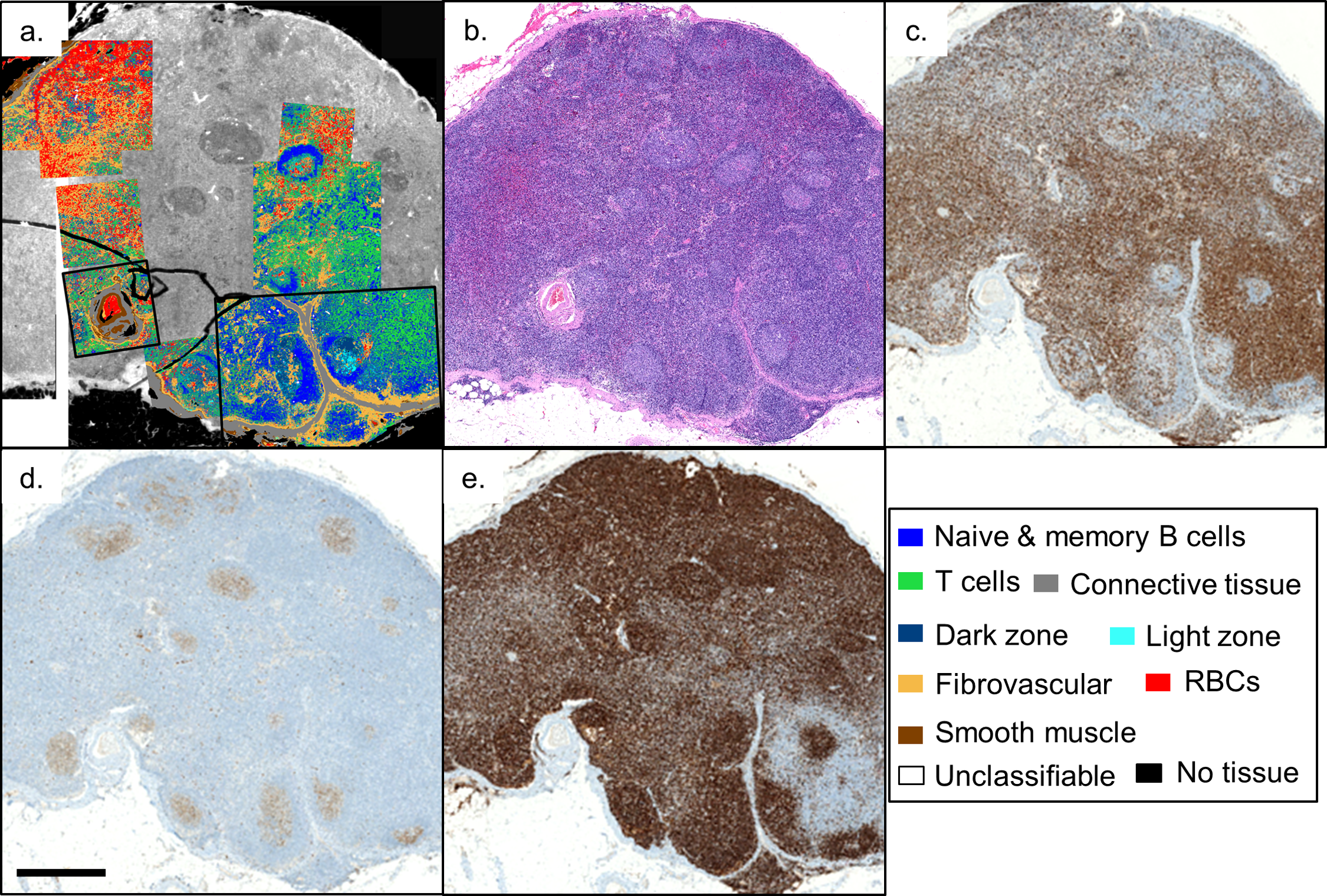

Supplement: S3 Fig — Eight classified HD FT-IR images of a healthy submandibular lymph node overlaid on a low-resolution FT-IR image at 1654 cm-1 band intensity, (a), and compared to serial sections of H&E, (b), CD3+, (c), CD10+, (d), and CD20+, (e), stains show that the classifier captures the global structure of the lymph node. The classified images with black outlines in (a) contain ROIs that were used to train the classifier (see Fig 2 and S1 Fig). The solid bar is 1 mm. (TIF) [file pone.0127238.s003.tif]

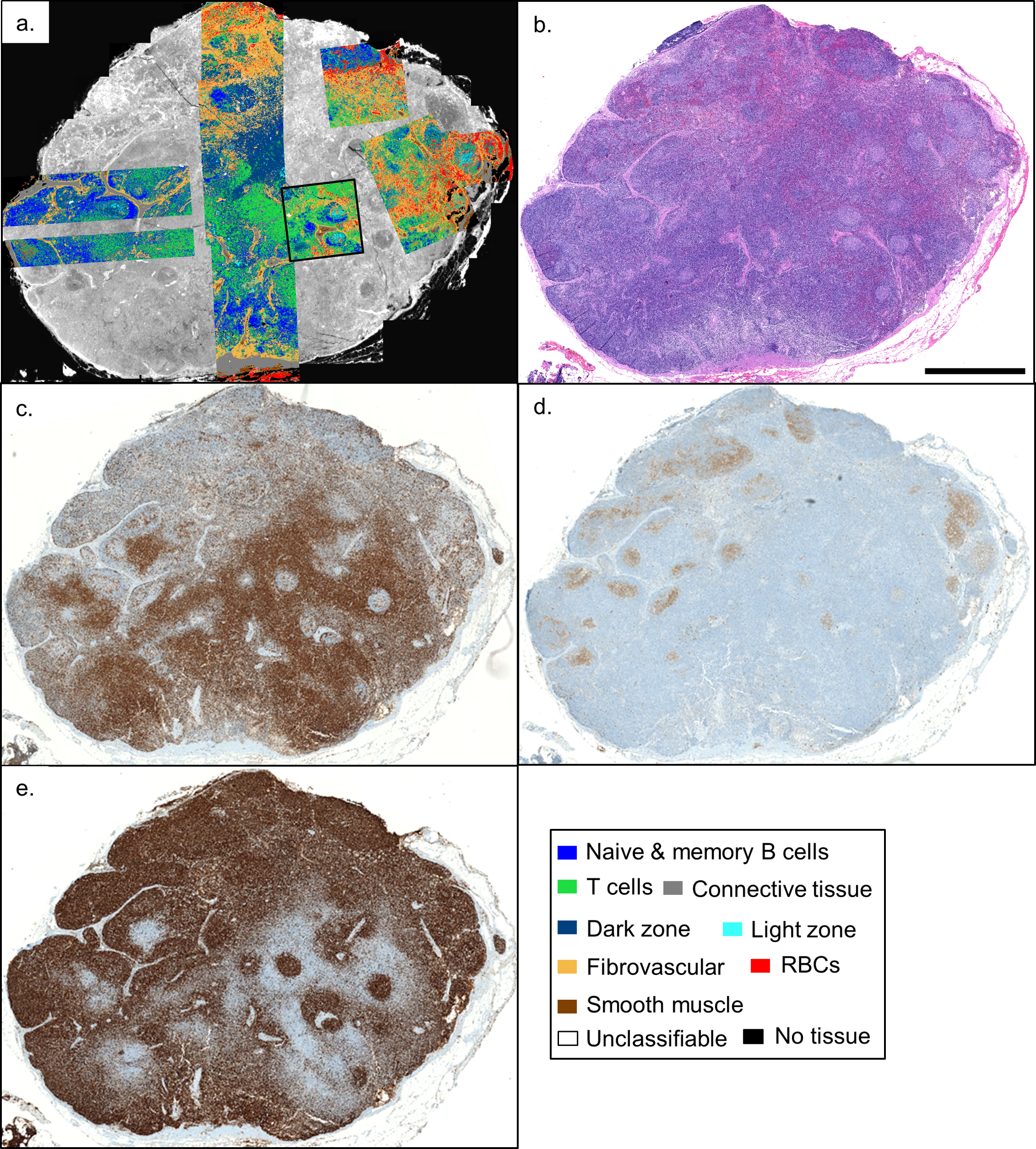

Supplement: S4 Fig — Seven classified HD FT-IR images of a healthy submandibular lymph node overlaid on a low-resolution FT-IR image at 1654 cm-1 band intensity, (a), and compared to serial sections of H&E, (b), CD3+, (c), CD10+, (d), and CD20+, (e), stains show that the classifier captures the global structure of the lymph node. The boxed classified image in (a) contains ROIs that were used to train the classifier (see Fig 2 and S1 Fig). The bar in (b) is 2 mm. (TIF) [file pone.0127238.s004.tif]

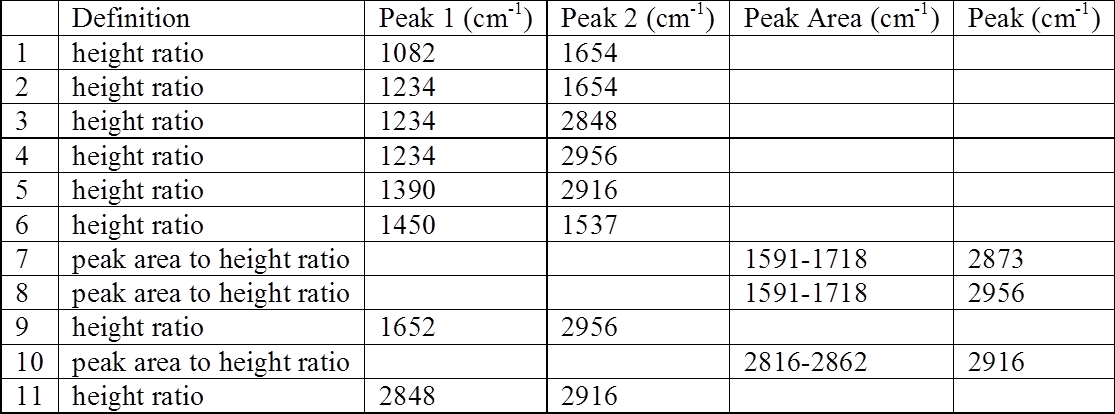

Supplement: S1 Table — (TIF) [file pone.0127238.s006.tif]
