# Supplementary material for: High Definition Infrared Spectroscopic Imaging for Lymph Node Histopathology
Source: PLoS One. 2015 Jun 3;10(6):e0127238. doi: 10.1371/journal.pone.0127238 (PMC4454651; doi:10.1371/journal.pone.0127238)
Supplement: S1 File — A secondary follicle in a healthy lymph node was imaged with an HD FT-IR instrument, (a), and a conventional system, (b). Corresponding HCA results with 10 classes are shown in (c) and (d), respectively. IR images are of 2900 cm-1 band intensity as indicated by the bar, and the solid bar is 200 microns. Fig. B. Effect of pixel binning on HCA. HCA results with 10 classes performed on the same HD IR image from a healthy submandibular lymph node at different levels of pixel averaging: (a) 20x, (b) 10x, (c) 5x, (d) 3x, (e) 2x and (f) 1x (original HD image). The solid bar is 400 microns. Fig. C. Binning effects on HCA. HCA results with 10 classes and 2x binning, performed on the image shown in Fig. A in S1 File with two different distance measures: D-Values and Euclidean located on the top and bottom rows, respectively. The left column is the original HD data, while the right column presents results from 2x-binned HD data. The solid bar is 200 microns. (DOCX) [file pone.0127238.s005.docx]

**Supplemental Material**


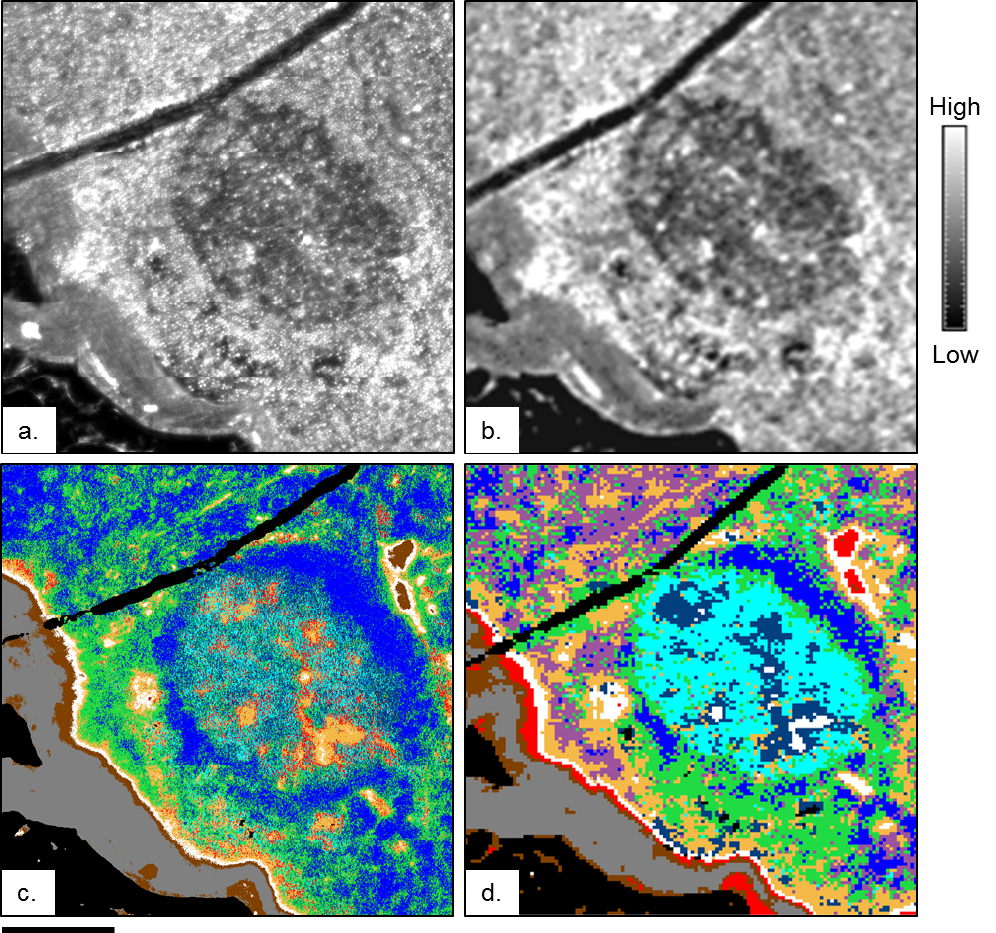


Fig. A in S1 File. Comparison of HCA performed on HD and low-res IR images. A secondary follicle in a healthy lymph node was imaged with an HD FT-IR instrument, (a), and a conventional system, (b). Corresponding HCA results with 10 classes are shown in, (c) and (d), respectively. IR images are of 2900 cm^-1^ band intensity as indicated by the bar, and the solid bar is 200 microns.

A comparison between Hierarchical Cluster Analysis (HCA) performed on HD and conventional IR images (Fig. Ac and Fig. Ad, respectively) demonstrates that the larger scale features such as the outer fibrous capsule (grey), the naïve B cell mantle (blue), and the location of the germinal center (region inside of blue ring) are distinguished in both cases, albeit with the expected increase in spatial quality in HD images. However, in the more heterogeneous regions of the tissue, the smaller image pixel size appears to actually hinder cellular assessment with most of the neighboring pixels assigned to different classes. This creates the “grainy” appearance of the HCA from the HD data, which is not present in the conventional IR dataset. Since the SNR of spectra from both conventional and HD instrumentation is nearly the same (see Fig. 1 d and e), we hypothesized that the source of this confounding result with HCA likely rests in the small pixel size. While the effect of large pixels has previously been examined[32], the effects of a sub-cellular pixel size on tissue recognition has not been reported. To investigate the performance of HCA at different pixel sizes, an HD IR image with two secondary follicles was down-sampled by averaging the neighboring pixels and then subjected to HCA – the results of which are shown in Fig. B in S1 File. While tissue can be segmented at each effective pixel size, the hazards of using HCA in the particular case of HD pixels can be seen in Fig. Bf in S1 File. The results, while largely segmenting the tissue in all cases, seem to be very heavily dependent on pixel size. It is not possible to confidently state which classification of tissue provides the most accurate result or if any one is superior to the other. To confirm that this behavior is not image specific, the HD IR image in Fig. A in S1 File was also averaged and is shown in Fig. C in S1 File.


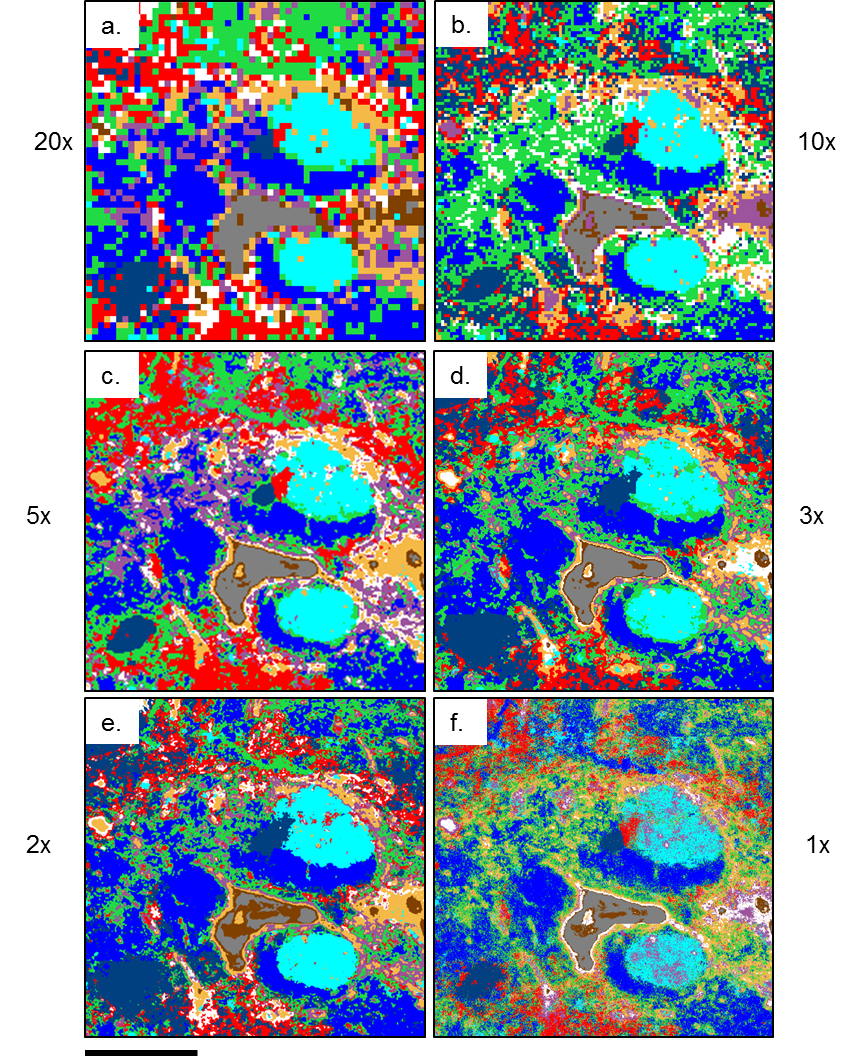


Fig. B in S1 File. Effect of pixel binning on HCA. HCA results with 10 classes performed on the same HD IR image from a healthy submandibular lymph node at different levels of pixel averaging: (a) 20x, (b) 10x, (c) 5x, (d) 3x, (e) 2x and (f) 1x (original HD image). The solid bar is 400 microns.


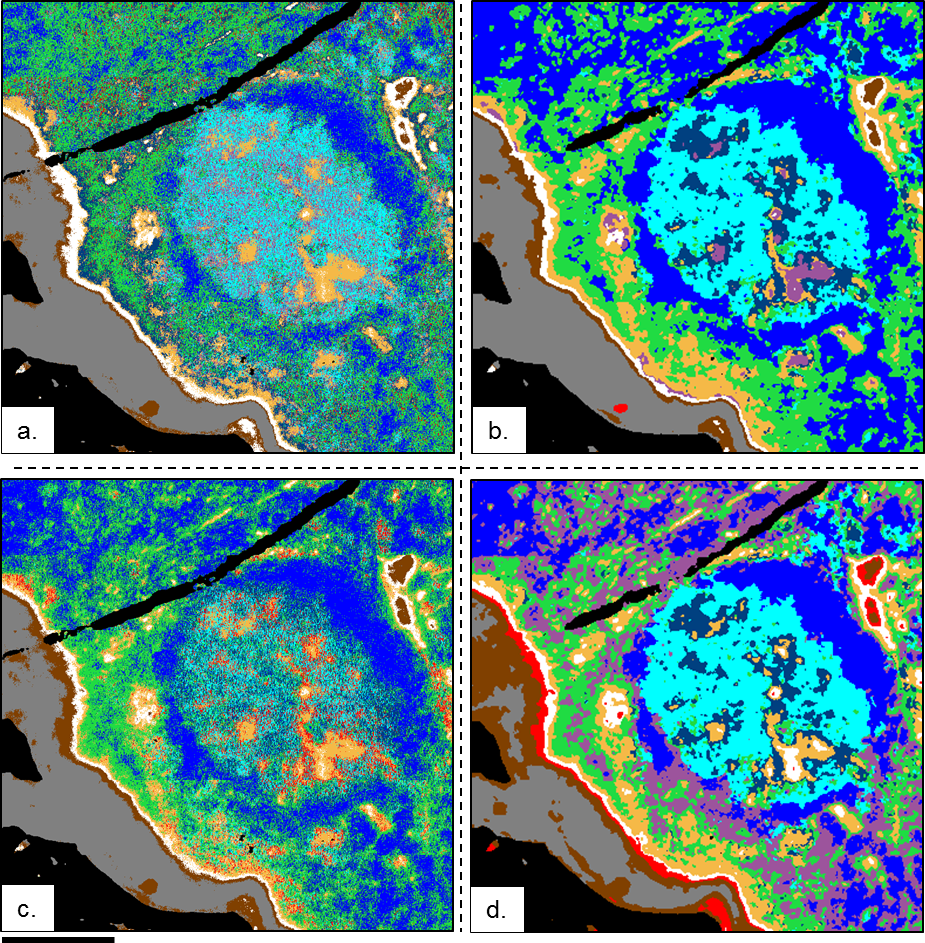


Fig. C in S1 File. Binning effects on HCA. HCA results with 10 classes and 2x binning, performed on the image shown in Fig. A in S1 File with two different distance measures: D-Values and Euclidean located on the top and bottom rows, respectively. The left column is the original HD data, while the right column presents results from 2x-binned HD data. The solid bar is 200 microns.


A further complication of HCA for tissue classification applications is that it is computationally intensive. As a result, in addition to pixel aggregation, analysis is typically restricted to a subset of the “fingerprint region” of the IR absorbance spectrum, discarding many peaks which could potentially aid in the segmentation of tissue and in distinguishing various cell types. For example, the HCA images shown in Figs. A-C in S1 File used only the 940-1265 cm^-1^ region and were at the edge of the current computational capabilities of a standard desktop, mostly due to limitations in operating memory. Additionally, while HCA may be initially unsupervised, whenever the resulting clustering is not as desired or expected, usually a different set of parameters (spectral range, distance measure, preprocessing) is used in order to obtain the expected result – in this manner the analysis ceases to be unsupervised.
